# Should glutamatergic modulators be considered preferential treatments for adults with major depressive disorder and a reported history of trauma? Conceptual and clinical implications

**DOI:** 10.1017/S1092852925100278

**Published:** 2025-05-26

**Authors:** Kayla M. Teopiz, Heidi K.Y. Lo, Moiz Lakhani, Angela T. H. Kwan, Poh Khuen Lim, Melanie Zhang, Sabrina Wong, Gia Han Le, Jennifer Swainson, Bing Cao, Christine Dri, Roger Ho, Kyle Valentino, Roger S. McIntyre

**Affiliations:** 1 Brain and Cognition Discovery Foundation, Toronto, ON, Canada; 2 Institute of Medical Science, University of Toronto, Toronto, ON, Canada; 3 Department of Psychiatry, University of Hong Kong, Hong Kong, China; 4 Faculty of Medicine, University of Ottawa, Ottawa, ON, Canada; 5 Department of Psychiatry, University of Toronto, Toronto, ON, Canada; 6 Department of Pharmacology, University of Toronto, Toronto, ON, Canada; 7 Department of Psychiatry, University of Alberta, Edmonton, AB, Canada; 8 Neuroscience and Mental Health Institute, Edmonton, AB, Canada; 9 Key Laboratory of Cognition and Personality, Faculty of Psychology, Ministry of Education, Southwest University, Chongqing, P. R. China; 10 Institute for Health Innovation and Technology (iHealthtech), National University of Singapore, Singapore; 11 Division of Life Science (LIFS), Hong Kong University of Science and Technology (HKUST), Hong Kong, China

**Keywords:** major depressive disorder, trauma, childhood maltreatment, glutamate, inflammation, ketamine, N-methyl-D-Aspartate Receptor

## Abstract

Major depressive disorder (MDD) is a chronic, highly prevalent, and debilitating mental disorder associated with significant illness and economic burden globally. Exposure to trauma (eg, physical, sexual, emotional abuse, and/or physical, and emotional neglect) is common among individuals with MDD. Persons with MDD and a history of trauma often exhibit an attenuated response to conventional serotonergic antidepressants compared to those with non-traumatized depression. Emerging evidence indicates that exposure to trauma is associated with increased inflammatory markers [eg, C-reactive protein (CRP), interleukin-6 (IL-6), tumor necrosis factor-α (TNF-α)] as well as glutamatergic dysregulation in the central nervous system (CNS). It is hypothesized that individuals with MDD and a history of trauma may be conceptualized as a distinct bio-phenotype compared to non-traumatized depression. Furthermore, preliminary evidence positions select glutamatergic modulators as potential, novel, mechanistically-informed therapeutic strategies that may provide benefit to persons with elevated inflammation and glutamatergic dysregulation.

The majority of adults with major depressive disorder (MDD) fail to achieve symptomatic or syndromal recovery with conventional first-line antidepressants.^1^ The aforementioned deficiency in extant treatment performance has provided the imperative to identify baseline sociodemographic, biologic, treatment, and contextual factors that are predictive of treatment response to antidepressants. A triangulation of evidence has not only described a high rate of trauma in persons with lived depressive experience, but also evidenced that a history of trauma results in a bio-phenotype of depression with reproducible symptomatic and neurobiological features.^2^^,^^3^ This perspective provides a succinct rationale for introducing a hypothesis that glutamatergic-signaling modulators may be preferred antidepressants in persons presenting with MDD. This perspective is not intended to be a review article of antidepressant outcomes as a function of trauma history, biomarkers, predictors of antidepressant response, or the neurobiology of glutamatergic-signaling modulators, but instead it is meant to provide a perspective and synthesis of the topic, as these reviews are published elsewhere.^4^^–^^6^ In addition, we are not specifically describing the influence of post-traumatic stress disorder (PTSD) or complex PTSD on antidepressant outcomes, but instead delimit our perspective to MDD with a history of trauma.

Major depressive disorder is a chronic, highly prevalent, and debilitating mental disorder associated with significant illness and economic burden globally.^7^ According to the Global Burden of Disease study in 2021, depressive disorders were associated with a substantial increase in disability-adjusted life-years (DALYs), highlighting the need for effective treatment strategies.^8^ However, it is estimated that one-third of individuals with MDD fail to achieve a clinically meaningful response to conventional serotonergic antidepressants.^1^ Moreover, individuals with a history of trauma are highly likely to exhibit an attenuated response to conventional serotonergic antidepressants.^4^

Trauma has been broadly defined as physical, sexual, or emotional abuse, as well as physical and emotional neglect.^4^^,^^9^ In addition, psychological trauma is an inclusive term that may overlap with the foregoing definition of trauma, and is operationalized as any stressful event that causes distress that exceeds an individual’s ability to cope with the emotional and/or cognitive response to the stressful experience.^10^ A history of trauma is common in individuals with MDD.^9^^,^^11^^,^^12^ In the International Study to Predict Optimized Treatment for Depression (iSPOT-D), which included 1008 adults with MDD and 336 matched healthy controls, participants with MDD had a fourfold or higher rate of childhood abuse compared to healthy controls.^4^ It is notable that trauma exposure and/or other specified trauma is dissociable from PTSD, as not all traumatic experiences (as well as symptoms as a result of trauma exposure) will result in the onset of PTSD.^13^^,^^14^ The prevalence of MDD and comorbid PTSD is amply documented in prior comprehensive reviews.^15^^–^^17^

Convergent evidence supports that MDD with a history of trauma may be conceptualized as a distinct bio-phenotype compared to non-traumatized depression.^1^ For instance, individuals with trauma-related MDD report different illness characteristics (e.g., increased suicidal behavior) and treatment outcomes (e.g., attenuated response to serotonergic antidepressants) compared to MDD without a history of trauma.^1^^,^^18^ In addition, findings from extant literature further suggest that exposure to trauma during critical developmental periods correlates with deficits in cognitive functions (e.g., processing speed, attention, and executive functioning) and reward-related processes in persons with MDD, highlighting an overlap in the neurobiological and psychological impact of trauma.^19^^–^^22^ Specifically, it has been reported that trauma in childhood is associated with increased vulnerability to stress, cognitive deficits, changes in brain structure, and disruptions in immune and metabolic functions in persons with MDD.^4^^,^^22^^–^^28^

Replicated evidence indicates that individuals with trauma-related depression experience an attenuated response to conventional serotonergic antidepressants compared to those with non-traumatized depression.^1^ For example, the iSPOT-D study reported that participants who experienced abuse before the age of seven, rather than those with a history of traumatic events in general, had significantly less improvement in treatment outcomes of both clinician- and self-rated depressive symptoms after eight weeks of serotonergic antidepressant treatment (i.e., sertraline) in adulthood.^4^ In addition, meta-analytic data from 10 clinical trials corroborated that individuals with MDD and a history of childhood maltreatment exhibited poorer response not only to monotherapy but also to combination therapy and psychotherapy.^29^

Emerging evidence supports that exposure to trauma is associated with increased inflammatory markers [e.g., C-reactive protein (CRP), interleukin-6 (IL-6), tumor necrosis factor-α (TNF-α)] in persons with depression.^9^^,^^11^ A meta-analysis reported that elevated CRP, IL-6, as well as select studies reporting a composite measure of inflammation [including CRP, IL-6, fibrinogen, E-Selectin, Intercellular Adhesion Molecule-1 (ICAM-1), and TNF-α], significantly mediated the association between adverse childhood experiences (ACE) and depression severity in adulthood.^11^ Currently, there are no established biomarkers to inform antidepressant treatment selection in individuals with MDD.^30^ However, the foregoing observations provide the rationale to hypothesize that differences in antidepressant response in persons with trauma-related depression may in part be attributed to neurobiological mechanisms underlying the relationship between elevated inflammation and history of trauma in persons with MDD.^11^

It is reported that alterations in stress, notably inflammation, are highly associated with glutamatergic dysregulation in the CNS.^31^ The mechanisms underlying the foregoing association are not fully characterized; however, it has been hypothesized that glutamate dysregulation is a consequence of pro-inflammatory effects on glial function in the CNS.^31^ It has been further proposed that inflammatory cytokines are associated with a decrease in glutamate transporter expression on astrocytes, as well as an increase in astrocytic glutamate release.^32^ As a result, excess glutamate in the extrasynaptic space is associated with increased N-Methyl-D-Aspartate (NMDA) receptor overactivation, excitotoxicity, and a decrease in brain-derived neurotrophic factor (BDNF) (Figure 1).

**Figure 1.**
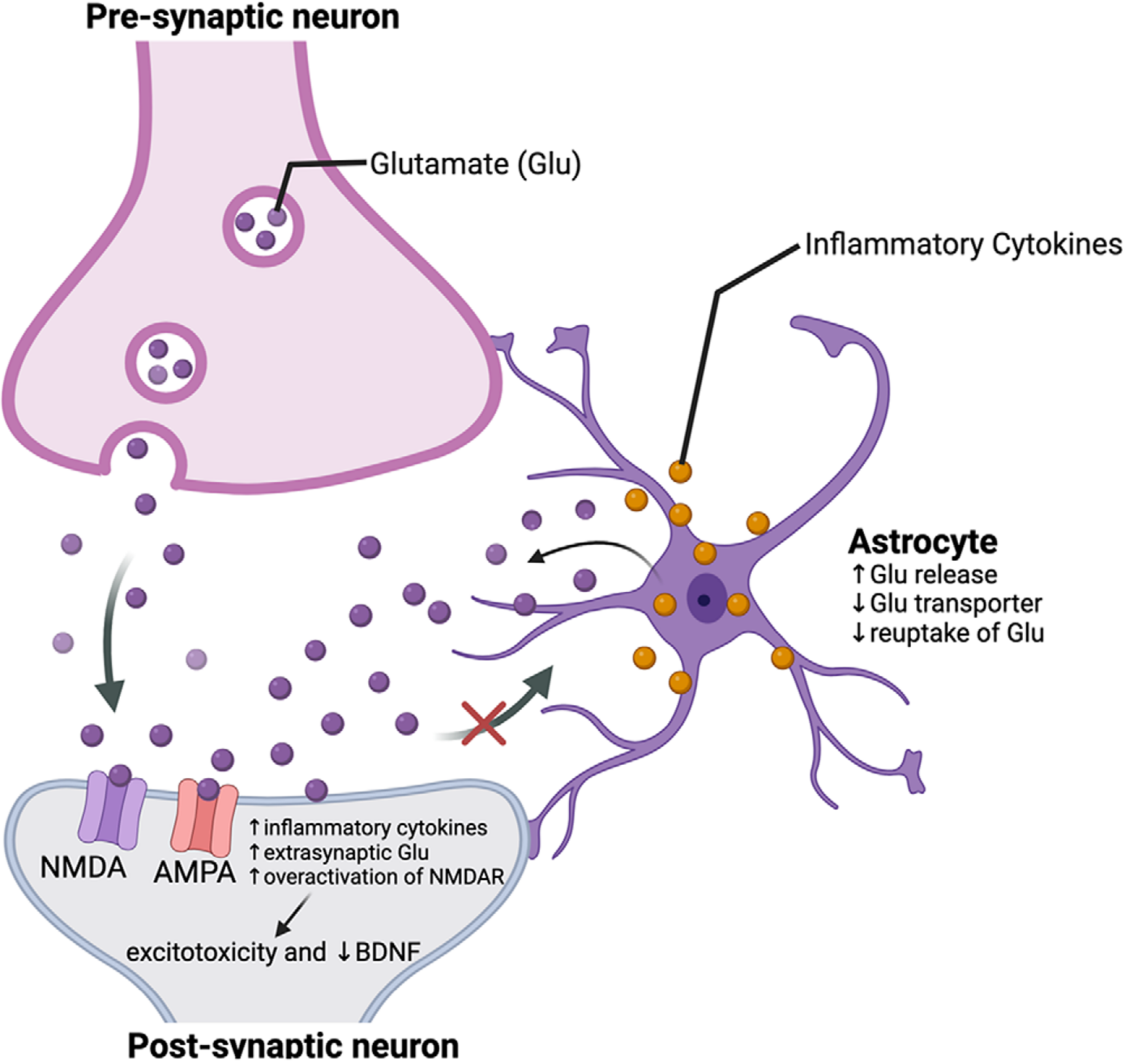
Interaction between inflammatory cytokines, glutamatergic dysregulation, and brain-derived neurotrophic factor (BDNF). Abbreviations: NMDA = N-Methyl-D-Aspartate receptor; AMPA = α-amino-3-hydroxy-5-methyl-4-isoxazolepropionic receptor; GLU = glutamate; BDNF = Brain-derived neurotrophic factor.

Replicated results from preclinical studies have documented an association between inflammatory markers, alterations in glutamate activity, and depression. For example, in a rat model of depression [i.e., the Flinders sensitive line (FSL)], it was observed that depressive-like behavior was associated with dysfunctional glutamatergic regulation, including downregulated glial glutamate transporter (GLAST).^34^

Additionally, preliminary evidence from human clinical studies suggests that inflammation is associated with glutamatergic dysregulation in disparate brain regions that may be implicated in depressive symptomatology. For example, administration of the inflammatory cytokine interferon alpha (IFN-α) was associated with increased glutamate in the left basal ganglia and dorsal anterior cingulate cortex.^35^ In a separate study conducted by the same research group involving outpatients with MDD, it was observed that increased CRP was significantly associated with increased glutamate in the left basal ganglia.^31^ Moreover, increased glutamate in the left basal ganglia has been associated with increased measures of anhedonia.^31^ Taken together, it could be hypothesized that an increase in inflammatory markers as a result of stress may subserve neurobiological mechanisms underlying the association between excess glutamate and heightened depressive psychopathology.^31^^,^^36^^,^^37^ Against this background, it is hypothesized that glutamatergic modulators may serve as a mechanistically-informed treatment option for persons with MDD and elevated inflammation and glutamatergic dysregulation.^38^

The United States Food and Drug Administration (FDA) has approved two glutamatergic modulators in the treatment of MDD. In 2019, intranasal esketamine (Spravato®) was FDA-approved in the treatment of adults with treatment-resistant depression (TRD) (commonly operationalized as failure of two or more adequate trials of antidepressants), as well as adults with MDD and acute suicidal ideation in 2020 (Table 1).^39^^,^^40^ Additionally, an oral combination of dextromethorphan-bupropion (AXS-05, Auvelity®) was FDA-approved in adults with MDD in 2020 (Table 1).^41^ Furthermore, a number of other glutamatergic modulators are being investigated in the treatment of MDD (e.g., ketamine, TAK-653, TS-161).^42^ However, there is a paucity of studies that have aimed to specifically evaluate the effect of trauma as a moderator or mediator of treatment response with a glutamatergic modulator in persons with MDD.^31^^,^^33^

**Table 1.** FDA-Approved Glutamatergic Antidepressants in Depression

FDA-Approved Glutamatergic Antidepressants
Esketamine adjunctive therapy in Adults with Treatment-Resistant Depression (2019) Esketamine adjunctive therapy in Adults with MDD and Suicidal Ideation (2020) NMDA receptor antagonist and sigma–1 agonist Intranasal administration
Dextromethorphan-bupropion combination in Adults with MDD (2022) NMDA receptor antagonist and sigma–1 receptor agonist Oral extended-release tablet

*Abbreviations:* FDA = United States Food and Drug Administration; NMDA = N-Methyl-D-Aspartate; MDD = major depressive disorder.

Ketamine, a noncompetitive antagonist of the heterotetrameric NMDA receptor and a glutamatergic modulator, has established efficacy in the off-label treatment of persons with depression, notably in persons who do not achieve syndromal or functional recovery with conventional antidepressants.^42^^–^^44^ In contrast to serotonergic antidepressants, ketamine has been reported to elicit a more robust treatment response in persons with MDD who have a history of trauma.^45^^,^^46^ For example, a growth mixture modeling (GMM) analysis of a sample of adults receiving intravenous (IV) ketamine for depression (n = 328) in a community clinic in 2021 identified three distinct patient groups based on ketamine treatment trajectory [measured by change on the Quick Inventory of Depressive Symptomatology—Self-Report (QIDS-SR)]: severe depression with rapid improvement, severe depression and minimal improvement, and moderate depression with gradual improvement.^45^ Post-hoc analyses of the foregoing study revealed that childhood physical abuse, as measured by the Childhood Trauma Questionnaire (CTQ), was the only pretreatment characteristic that significantly differed between the severe depression with rapid improvement group and the severe depression with minimal improvement group (i.e., reported childhood physical abuse in persons with severe depression was significantly associated with rapid improvement with ketamine).^45^ The aforementioned findings highlight the value of identifying pretreatment characteristics that may moderate or mediate treatment response in a sample of persons with severe depression exhibiting distinct and divergent treatment trajectories.^45^

An additional latent class analysis was conducted by the same research group on a new sample of patients with depression (n = 298) receiving IV ketamine treatment.^46^ It was observed that pretreatment characteristics, notably childhood trauma, are associated with rapid improvement in depressive symptoms with ketamine treatment in persons with severe depression compared to persons with moderate depression.^46^ The foregoing finding highlights that childhood trauma may moderate the trajectory of treatment response in a subgroup of persons with depression, further providing the impetus to investigate the mechanisms that subserve improved (or poorer) treatment outcomes with ketamine. For example, it has been hypothesized that ketamine, and potentially other glutamatergic antagonists, block the development of sensitization to exposure to stress and/or trauma.^46^

In addition, there is preliminary evidence of select anti-inflammatory and neurobiological biomarkers that are associated with antidepressant response to ketamine. For example, it was observed that pretreatment inflammatory markers predict antidepressant response in persons receiving IV ketamine for depression.^43^ Further preliminary evidence suggests that select electroencephalographic measures are associated with response to ketamine and esketamine in persons with TRD, commonly operationalized as failure of two or more adequate trials of antidepressants.^1^^,^^6^ A separate study reported that a history of early life stress was associated with both altered gene expression in the nucleus accumbens and attenuated response to various classes of antidepressants in both humans and murine models.^47^ Moreover, early life stress was the strongest predictor of response to ketamine treatment in the foregoing murine model.^47^ However, it is noted that additional negative studies have not detected an association between history of trauma and response to ketamine, providing the impetus for future studies to explore history of trauma as a baseline moderator of treatment response in MDD.^48^

Additional lines of research indicate that vortioxetine, a multimodal antidepressant approved in the treatment of adults with MDD, is another example of an agent with glutamatergic modulation and anti-inflammatory effects that has demonstrated treatment efficacy in persons with trauma-related depression.^12^ In an analysis of four double-blind, randomized, placebo-controlled short-term studies, vortioxetine (5–20 mg/day) was reported to be highly effective in the treatment of persons with MDD and childhood or recent trauma.^12^ It was also reported that persons with MDD and past trauma who were assigned to vortioxetine treatment were significantly less likely to relapse compared to persons assigned to placebo.^12^ Taken together, it can be posited that the therapeutic effect of vortioxetine in persons with MDD and trauma may, in part, be attributable to its demonstrated effects on glutamate modulation and anti-inflammatory properties. However, the foregoing findings were observed from post-hoc analyses of four double-blind randomized controlled trials in adults with MDD, and thus, additional research that primarily aims to assess the efficacy of vortioxetine in the treatment of trauma-related MDD is warranted.

Convergent evidence further supports that vortioxetine, in contrast to selective serotonin reuptake inhibitors (SSRI), increases the firing of pyramidal neurons.^49^ It is hypothesized that the observed effect of vortioxetine on glutamatergic transmission is a result of the downstream effect of antagonism at the 5-HT3 receptors, thus blocking 5-HT-mediated inhibition of gamma-aminobutyric acid (GABA) interneurons and an overall disinhibition of glutamatergic pyramidal neurons.^49^ Moreover, vortioxetine has demonstrated anti-inflammatory effects, such as blocking neuroinflammation via activation of 5-HT2b and 5-HT7 receptors, resulting in inhibition of activated (M1) microglia to favor the anti-inflammatory (M2) microglia phenotype.^50^^,^^51^

Notwithstanding the distinction between exposure to trauma and PTSD, it is notable that separate lines of research have aimed to investigate the efficacy and effectiveness of select glutamatergic modulators in the treatment of PTSD. For example, a separate proof-of-concept, randomized, double-blind, crossover trial compared IV ketamine with an active control (i.e., midazolam) in adults with PTSD (n = 41). At 24 hours post-infusion, IV ketamine treatment was associated with greater and rapid reduction in PTSD symptom severity in comparison to midazolam.^52^ In addition, recent meta-analytic data suggest that ketamine pharmacotherapy is effective in reducing both PTSD and depressive symptom severity in affected individuals.^53^ However, it is also noted that in persons with TRD (i.e., persons who do not respond to two or more conventional antidepressants), their reported therapeutic response to IV ketamine was independent of comorbid PTSD, providing the basis to further explore the overlapping mechanisms that may explain differences in treatment outcomes in persons with both MDD and PTSD, compared to PTSD alone.^54^

In addition, lamotrigine, approved by the FDA as an anticonvulsant and maintenance treatment in persons with bipolar disorder, has been hypothesized to modulate glutamate via inhibition of voltage-gated sodium channels with downstream inhibitory effects on glutamate release.^55^^,^^56^ Lamotrigine was preliminarily studied in adults with PTSD (n = 14) in a 12-week, double-blind, randomized, placebo-controlled trial.^57^ Of the 10 participants who were assigned to lamotrigine treatment (25 mg/day), five (50%) reported benefit in PTSD symptoms compared to one out of four (25%) participants assigned to placebo.^57^

Preliminary results of another glutamatergic modulator, D-cycloserine, suggest potential therapeutic benefit in adults with PTSD receiving exposure therapy.^58^ For instance, a pilot, randomized, double-blind, placebo-controlled trial investigated augmentation of D-cycloserine (100 mg) or placebo combined with virtual reality exposure therapy in adults with chronic PTSD (n = 25)^59^. The foregoing study reported that the group assigned to virtual reality exposure combined with D-cycloserine exhibited more rapid and greater improvement in PTSD symptoms compared to the virtual reality exposure combined with placebo.^59^ It is hypothesized that D-cycloserine may target PTSD symptoms via partial agonism of the NMDA receptor, resulting in both increased glutamate signaling and enhanced extinction learning (wherein extinction learning is associated with reduced conditioned response to stimuli in models of PTSD).^59^^,^^60^ However, there are contradicting reports of combination treatment of D-cycloserine and exposure therapy that resulted in significantly less improvement in PTSD symptoms compared to exposure therapy with placebo.^61^ As a result, further investigation of the potential therapeutic effect of D-cycloserine, as well as the neurobiological mechanisms that may subserve psychopathology in trauma-related symptoms, is needed.

It is crucial to note that the foregoing preliminary evidence supports the therapeutic benefit of select glutamatergic modulators in the treatment of PTSD; however, these findings cannot be considered equivalent to treating trauma-related depression. Instead, the foregoing evidence lends inferential support to the hypothesis that glutamatergic modulation may be of transdiagnostic benefit in persons living with mental disorders characterized by a history of trauma. Moreover, we are not drawing a pharmacodynamic equivalence across the aforementioned agents (i.e., vortioxetine, ketamine/esketamine, lamotrigine, D-cycloserine). The mechanism of action across these agents is complex, unknown, and cannot be reduced to a singular pharmacodynamic mechanism.^62^^–^^65^ However, preclinical and pharmacological evidence has suggested that vortioxetine, ketamine/esketamine, lamotrigine, and D-cycloserine not only affect glutamatergic neuron activity directly or indirectly but may also have antidepressant efficacy relevant to the treatment of persons with trauma-related symptoms.^62^^–^^65^

Notwithstanding reports of an amplified therapeutic response to select glutamatergic modulators in persons with trauma-related depression, there are a number of methodological limitations that affect the interpretation and inference of the aforementioned findings. Firstly, available studies may not characterize all types of trauma within their sample (e.g., physical, sexual, or emotional abuse, physical or emotional neglect, as well as aspects of relational trauma within families).^66^ Secondly, studies may vary in the number and recency of traumas reported by participants (e.g., a single traumatic event compared to multiple events). Thirdly, participants may vary with respect to the age of exposure to trauma (e.g., early life stress and/or childhood trauma versus trauma in adulthood). Additionally, measures of trauma may vary across studies depending on the method of assessment (e.g., the CTQ). Furthermore, trauma events vary with respect to pre-existing psychopathology in the affected individual. The foregoing factors increase heterogeneity among persons who have experienced trauma and influence trauma reactions and sequelae such as PTSD. Overall, an overarching limitation of the perspective paper herein is that we cannot make a generalized statement indicating that all trauma, in all of its forms and occurrences, would be preferentially responsive to glutamatergic modulators. However, this level of refinement is a future research vista.

A translational limitation of the perspective herein is the relative lack of glutamatergic modulators for persons with depressive disorders. For example, only dextromethorphan–bupropion (AXS-05, Auvelity®) is FDA-approved in non-TRD populations.^42^ However, this agent may not be accessible, available, or affordable to many persons. In addition, notwithstanding that esketamine/ketamine are indicated for adults with TRD, they are not considered first-line treatments in MDD and are limited with respect to access and affordability.^67^

It is also noted that the selection and priority of an antidepressant are probabilistic rather than deterministic. Taken together, the confluence of neurobiological factors that are replicated as abnormal in association with trauma exposure significantly reduces the probability of an acute SSRI response and/or increases the probability of acute response to a glutamatergic modulator. Notwithstanding, it cannot be concluded that the selection or priority of glutamatergic modulators in such clinical presentations would be deterministic. Currently, there is no biomarker or biosignature that is capable of guiding antidepressant selection in persons with mental disorders.^68^^,^^69^ There also remains an absence of fit for purpose phenotypic characteristics that are meaningful and predictive of antidepressant treatment response. Extant literature indicates that persons with a history of trauma experience an attenuated response to serotonergic antidepressants.^4^ However, preliminary evidence suggests that there may not be an attenuated response to select glutamatergic modulators (e.g., ketamine) among persons with a history of trauma.^45^^,^^46^ Taken together, preliminary findings suggest that a history of trauma, as an anamnestic aspect, may be informative with respect to antidepressant selection.^11^ A priority future research vista includes randomized controlled studies that aim to evaluate the efficacy of a glutamatergic modulator compared to a serotonergic antidepressant in persons with a history of trauma. The results of the proposed investigation may inform algorithms for treatment selection and sequencing among persons with trauma-related depression, who may represent a distinct bio-phenotype (e.g., elevated inflammation and glutamate dysregulation).

## Data Availability

This manuscript does not present new data. All supporting literature and data referenced are publicly available and cited appropriately within the text.
